# Potential of Multivariate Statistical Technique Based on the Effective Spectra Bands to Estimate the Plant Water Content of Wheat Under Different Irrigation Regimes

**DOI:** 10.3389/fpls.2021.631573

**Published:** 2021-02-26

**Authors:** Hui Sun, Meichen Feng, Lujie Xiao, Wude Yang, Guangwei Ding, Chao Wang, Xueqin Jia, Gaihong Wu, Song Zhang

**Affiliations:** ^1^Institute of Dry Farming Engineering, Shanxi Agricultural University, Taigu, China; ^2^College of Resource and Environment, Shanxi Agricultural University, Taigu, China; ^3^Department of Chemistry, Northern State University, Aberdeen, SD, United States

**Keywords:** band selection, canopy reflectance, PLSR, transformation method, water content

## Abstract

Real-time, nondestructive, and accurate estimation of plant water status is important to the precision irrigation of winter wheat. The objective of this study was to develop a method to estimate plant water content (PWC) by using canopy spectral proximal sensing data. Two experiments under different water stresses were conducted in 2014–2015 and 2015–2016. The PWC and canopy reflectance of winter wheat were collected at different growth stages (the jointing, booting, heading, flowering, and filling stages in 2015 and the jointing, booting, flowering, and filling stages in 2016). The performance of different spectral transformation approaches was further compared. Based on the optimal pretreatment, partial least squares regression (PLSR) and four combination methods [i.e., PLSR-stepwise regression (SR), PLSR-successive projections algorithm (SPA), PLSR-random frog (RF), and PLSR-uninformative variables elimination (UVE)] were used to extract the sensitive bands of PWC. The results showed that all transformed spectra were closely correlated to PWC. The PLSR models based on the first derivative transformation method exhibited the best performance (coefficient of determination in calibration, *R*^2^_C_ = 0.96; root mean square error in calibration, RMSE_C_ = 20.49%; ratio of performance to interquartile distance in calibration, RPIQ_C_ = 9.19; and coefficient of determination in validation, *R*^2^_V_ = 0.86; root mean square error in validation, RMSE_V_ = 46.27%; ratio of performance to interquartile distance in validation, RPIQ_V_ = 4.34). Among the combination models, the PLSR model established with the sensitive bands from PLSR-RF demonstrated a good performance for calibration and validation (*R*^2^_C_ = 0.99, RMSE_C_ = 11.53%, and RPIQ_C_ = 16.34; and *R*^2^_V_ = 0.84, RMSE_V_ = 44.40%, and RPIQ_V_ = 4.52, respectively). This study provides a theoretical basis and a reference for estimating PWC of winter wheat by using canopy spectral proximal sensing data.

## Introduction

Climate change has increased the frequency and intensity of drought events. In most part of Asia, drought has been recorded to intensify during the last decades (Miyan, 2015), which raises numerous challenges in agriculture. Water is one of the important factors in plant growth and yield formation. With the increase of water deficit, crops, such as wheat, appear to be gradually wilted, and the rate of growth slows down, resulting in 20–40% production loss in cereal (Daryanto et al., 2017). Thus, accurate and real-time estimation of plant water status could help the planters to adjust the irrigation management effectively and efficiently.

The conventional way to measure plant water content is based on destructive field sampling and laboratory analysis, which is always time consuming and labor demanding. In contrast, spectral reflectance is considered as a rapid and nondestructive technique taken into practical application (Cho et al., 2007). Plant water condition directly influences cell turgor and internal space of tissue resulting in the changes of leaf structure. It causes the absorption, transmission, and reflection of light in the leaves changing the value of canopy reflection eventually. The near-infrared and short-infrared spectral regions are sensitive to the water content of plant leaf and canopy (Ihuoma and Madramootoo, 2017). Based on the theory, researchers proposed many vegetation indices to monitor water content, such as land surface water index (LSWI) (Xiao et al., 2005), simple ratio water index (SRWI) (Zarco-Tejada et al., 2003), normalized difference water index (NDWI) (Gao, 1996), and so on. Furthermore, plant water content affects chlorophyll content and canopy size. For this reason, previous findings indicated that bands in the visible region can be used indirectly to assess plant water status (Das et al., 2017; El-Hendawy et al., 2019a).

Hyperspectral remote sensing, having numerous continuous narrow bands and providing crucial information, has shown great potential for the accurate retrieval of plant parameters (Clevers et al., 2010). As for plant water status, there were approaches based on narrow band vegetation indices (Winterhalter et al., 2011; Wang et al., 2015; Zhang and Zhou, 2015; Fang et al., 2017) and spectral absorption features (Tian et al., 2001; Mutanga et al., 2005; Cheng et al., 2011). However, the canopy spectrum can generate comprehensive information which not only extracts the expression of the target variation but also other factors (e.g., soil background, noise of instrument) (Demetriades-Shah et al., 1990). In order to reduce useless information and improve the signal-to-noise ratio, the reflectance data are properly processed or transformed before the actual analysis. Numerous studies have indicated that different pretreatment methods are used to predict the water status of plant, including the first derivative (Liang et al., 2013), continuum removal (CR) (González-Fernández et al., 2015), and normalization (Sun et al., 2015). Moreover, the hyperspectral data contains a large number of bands (e.g., FieldSpec 3.0 Spectrometer has more than 1,900 bands), resulting in redundancy and multicollinearity. In the modeling process, it also makes the number of samples much smaller than the number of independent variables (spectral wavelengths) used in the spectral analysis (Atzberger et al., 2010). Partial least square regression (PLSR) is an effective method to solve these problems. PLSR, which combines principal component analysis and multiple linear regression, can easily process the data matrix and solve the correlation between independent variables. It has been used to estimate vegetation water content (Li et al., 2008; Mirzaie et al., 2014). However, the PLSR model with full spectrum is complicated in practical applications. In order to remove the irrelevant information, reduce the number of input variables, simplify the complexity of the model, and improve the interpretability of the model, it is necessary to select the sensitive wavelengths. There are already many variable selection methods. Zou et al. (2010) made a review about the variable selection methods in near-infrared spectroscopy. Some variable selection methods were used in plant water status, such as Bipls-SPA (backward interval PLS in combination with successive projection algorithm) (Zhang et al., 2012), PLSR-SR (Das et al., 2017), random frog (Chen and Li, 2020), and so on. These models established with sensitive bands also had good prediction ability.

As an important step in quantitative spectral analysis, data preprocessing had a significant influence on improving PLSR model performance. PLSR is proved to be a useful approach in selecting sensitive wavelengths (Sharabian et al., 2014; Wang et al., 2017; Zeng et al., 2018) and quantitating plant parameters. Considering the advantages of pretreatment, more sensitive bands would be selected from the spectra with optimal pretreatment. To date, few studies compared multivariate statistical methods in sensitive band selection based on the optimal pretreatment method. In this study, nine common transformation methods in combination with PLSR were initiated and compared to select the suitable transformation method of canopy reflectance from field spectrometer data for estimating plant water content in winter wheat. Meanwhile, in order to reduce the input variables of the PLSR model, sensitive wavelengths were selected by PLSR and four combined methods [i.e., PLSR in combination with stepwise regression (SR), successive projections algorithm (SPA), random frog (RF), and uninformative variables elimination (UVE)].

## Materials and Methods

### Site Description and Experimental Design

The experiment was conducted from 2014 to 2016 at the experiment station of Shanxi Agricultural University (E112°34′19.96″, N37°25′19.81″), Shanxi Province (P. R. China). The experimental site has a temperate continental climate condition. The average annual temperature is 9.8°C, the annual frost-free season is around 175 days, and the annual precipitation is around 450 mm.

Experiments were conducted in a water-experiment pool. The refilled soil in the pool is classified as Calcareous Cinnamon soil (Alfisols in US taxonomy) with 9.60 g/kg organic matter, 57.75 g/kg available nitrogen, 22.10 mg/kg available phosphate, and 185.48 mg/kg available potassium. The density of the soil is 1.36 g/cm^3^. The field capacity (FC) is 24.14%.

Jinnong 190 and Chang 4738 were planted in 2014–2015, while Chang 4738 and Zhongmai 175 were studied in 2015–2016. The experiments were set up in a randomized complete block design with three replications. There were five upper limits of irrigation for each 10-day period included in 2014–2015: W_1_ (80% of the FC), W_2_ (60% of the FC), W_3_ (45% of the FC), W_4_ (35% of the FC), and W_5_ (less than 30% of the FC). There were five irrigation regimes included in 2015–2016: I_1_ (four irrigations at jointing, booting, flowering, and filling stage), I_2_ (three irrigations at jointing, booting, and filling stage), I_3_ (two irrigations at jointing and flowering stage), I_4_ (two irrigations at jointing and filling stage), and I_5_ (without irrigation). The upper limit in 2015–2016 was 80% of the FC. The volume of water was controlled by the water meter. The plot area was 6 m^2^. The critical growth stages of wheat were selected based on the information available from the previous studies (Zadoks et al., 1974). For all treatments, the fertilizer was applied as basal dose with pure nitrogen (urea) 150 kg/ha, P_2_O_5_ 150 kg/ha, and K_2_O 150 kg/ha.

### Canopy Reflectance Measurement

The canopy spectral reflectance was captured from jointing stage to filling stage with a FieldSpec 3.0 Spectrometer [Analytical Spectral Devices (ASD), Boulder, CO, United States]. The sample dates are listed in Table 1. The spectral range of the device is 350–2,500 nm, with a sampling interval of 1.4 nm and spectral resolution of 3 nm between 350 and 1,000 nm and a sampling interval of 2 nm and spectral resolution of 10 nm between 1,000 and 2,500 nm. The measurements were conducted under clear sky conditions during 10:00–14:00. The sensor with a field of view of 25° was held at a height of 1 m above the canopy vertically to cover a sensing area of the wheat canopy (∼44.4 cm in diameter). In order to reduce the random error, spectral measurements were determined at three sites in each plot. Ten reflectance curves per site were averaged. A 40-cm^2^ BaSO_4_ calibration panel was used for calibrating the baseline reflectance prior to each measurement.

**TABLE 1:** Field reflectance spectra measurement dates in 2015 and 2016.

Year	Date 1	Date 2	Date 3	Date 4	Date 5
2015	13 April	24 April	3 May	13 May	22 May
2016	13 April	22 April	–	13 May	21 May

### Plant Water Content Measurement

Samples were collected consistent with the spectral measurements, i.e., 20 plants were clipped at ground level in each plot and immediately put into valve bags avoiding water loss. Then, the fresh plant was weighed. Samples were dried in an oven at 105°C for half an hour, then dried at 80°C to constant weight and reweighed. Plant water content (PWC) was calculated as Equation (1):

(1) $$PWC\left(\%\right)=\frac{FW-DW}{DW}\times 100\%$$

Where FW is the fresh weight of wheat (g), and DW is the dry weight of wheat (g).

### Spectral Transformation Methods

In order to get rid of the instrument noise, a moving Savitzky–Golay filter (Savitzky and Golay, 1964) with a window width of 5 nm and a polynomial of second degree was applied. The spectrum from 400 to 2,450 nm was selected. Due to the strong influence of the water vapor absorption peak on the canopy reflectance, wavelengths around 1,400 (1,350–1,400 nm) and 1,900 nm (1,800–1,950 nm) were removed.

**FIGURE 1:**
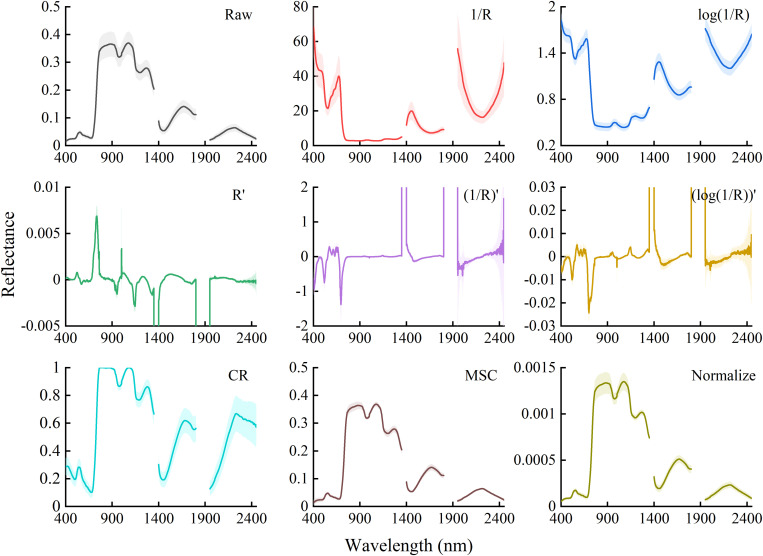
Average reflectance spectra and their standard deviations of the raw spectra and different transformed spectra. *R* is the raw reflectance of winter wheat. *R*′ is first derivative of *R*. CR, MSC, and Normalize represent the transformations of raw reflectance by continuum removal, multiplicative scatter correction, and normalization, respectively.

According to previous studies, nine transformation methods were conducted on the canopy reflectance to improve the accuracy and select the best method in evaluating the PWC of winter wheat. The transformation methods are listed in Table 2, and the raw spectrum and the transformed spectra are shown in Figure 1.

**TABLE 2:** Transformation methods used in this study.

No.	Transformation	Abbreviation	References
1	Raw reflectance	_*R*_	
2	The reciprocal reflectance	_1/*R*_	
3	Logarithm of the reciprocal reflectance	_*Log*(1/*R*)_	Grossman et al. (1996)
4	First derivative reflectance	_*R’*_	Demetriades-Shah et al. (1990)
5	First derivative of the reciprocal reflectance	_(1/*R*)′_	
6	First derivative of logarithm of the reciprocal reflectance	_(*Log*(1/*R*))′_	
7	Continuum removal	CR	Clark and Roush (1984)
8	Multiplicative scatter correction	MSC	Liang et al. (2010)
9	Normalization	Normalize	Yu et al. (1999)

### Partial Least Squares Regression

Partial least squares regression is one of the multivariate statistical analysis methods, which is a powerful tool in chemometrics and other fields (Wold et al., 1983). PLSR can handle data with high dimensionality and multicollinearity by reducing extensive collinear variables to noncorrelated factors (latent variables, LVs) (Wold, 1966). Then, the estimation model was established with LVs as the independent variables. In addition, the model is a linear regression model by projecting the independent variables and dependent variables (observable variables) to a new space. The method summarizes the spatial change information of independent variables, which can interpret the dependent variable as much as possible. In order to avoid overfitting, models were validated by leave-one-out cross-validation, and the RMSE_CV_ was a basic criterion of determining the number of the latent variables. In general, the optimal latent variable number of the PLSR model was determined by the minimum RMSE of cross-validation, which was the inflection point or the location where the curve of RMSE_CV_ became smooth (Kohonen et al., 2009).

### Selection of Sensitive Bands

The aim of selecting sensitive bands is to extract the informative bands, simplify the complexity of the model, and construct a stable PWC estimation model. Based on a PLSR model, regression coefficients (called *B*-coefficients) and the variable importance in the projection (VIP) were commonly used as variable selection methods. In this study, five selection methods were used. In these methods, the first step was to selected the band ranges by the PLSR regression coefficients (*B*-coefficients) and the variable importance in the PLSR projection (VIP). *B*-coefficients represent the importance of each band in predicting the dependent variable. VIP delineates the relative importance of each band in the PLSR model for predicting PWC. The VIP method selects variables by calculating the VIP score for each variable and excludes all the variables with VIP score below threshold 1 (Maestre, 2004). The VIP value for band *j* was calculated by the equation (Tran et al., 2014):

(2) VIPj=p×∑m=1Mwmj2SS(b⋅mt)m∑m=1MSS(bm⋅tm)

Where *p* is the number of bands, *M* is the number of selected LVs, *w*_*mj*_ is the corresponding loading weight (the linear combination of the independent variables that define the LVs) of the *j*-th band for the *m*-th latent variable, and SS(*b_*m*_⋅t_*m*_*) is the explained sum of squared *Y* (PWC) by the PLSR model with *m*-th latent variable.

More effective bands could be selected through combining the *B*-coefficients and the VIP value in band selection (Chong and Jun, 2005). However, the combination method may select some bands with false positive (Tran et al., 2014). Thus, it is necessary to combine with other band selection methods. Four other variable selection methods were studied.

#### Stepwise Regression

Stepwise regression is a common method of selecting variables. The aim of the regression is to screen for significant bands by establishing the relation between the dependent variables and the independent variable. In this study, bidirectional elimination that combined forward and backward elimination was used. It is an iterative process starting with no band. In each step, the band that contributes the most in predicting the dependent variable will be added to the model for a criterion of *P* value (*P* < 0.01). Then, the *P* value of all bands in the model will be tested, and bands whose *P* value is above the certain threshold (0.05) are removed. The process will repeat until all significant bands are in the regression model in estimating the dependent variable.

#### Successive Projections Algorithm

Successive projections algorithm is a method to solve the collinearity problem among variables (Araújo et al., 2001). It used a simple projection operation to obtain subsets of variables with minimal collinearity and is a forward algorithm for band selection. A new selected variable had the maximum projection value on the orthogonal subspace of the previous selected variable. The sensitive bands and the number of sensitive bands were determined based on the smallest root mean square error (RMSE) of cross-validation of multiple linear regression model.

#### Random Frog

Random frog is an efficient method for band selection in recent years. It was based on the framework of inversible-jump Markov Monte Carlo. In this method, PLSR was used as a modeling method. According to definite criteria, the variable subsets are updated constantly. When the number of iterations is reached, the selected frequency of each variable was calculated as the basis for eliminating redundant information (Yun et al., 2013). It requires a threshold of probability which is usually set empirically. Thus, a forward variable selection was used. According to frequency, bands were ranked from the largest to the smallest. In each iteration, the top-ranked band was added into the PLSR model. With the leave-one-out cross-validation, the number of bands was decided when the minimum RMSE was reached.

#### Uninformative Variables Elimination

Uninformative variables elimination is proposed to eliminate uninformative variable (Centner et al., 1996). Bands were evaluated with the stability coefficients of the regression coefficient (*B*-coefficient). A same size matrix of noise was jointed with the spectra matrix. The PLSR prediction model was established by the new independent variable matrix. Having leave-one-out validation, the *B*-coefficient matrix with sample in row and variable (band and noise) in column was obtained. Then, the stability coefficients were calculated with the average value divided by the standard deviation value of each variable *B*-coefficient. Compared with noise variable, the band with lower absolute stability coefficient would be removed.

### Performance Evaluation of the Developed Models

The experiment data collected from 2014 to 2015 (*n* = 150) and from 2015 to 2016 (*n* = 120) were used for model calibration (C) and validation (V), respectively. The PLSR models for estimating the PWC were evaluated based on the coefficient of determination (*R*^2^), RMSE, and ratio of performance to interquartile distance (RPIQ). The calculation formulae are listed as Equations 3–5:

(3) $$R^{2}=1-\frac{\sum_{i=1}^{n}{\left({\text{y}}_{\text{i}}-{\tilde{\text{y}}}_{\text{i}}\right)}^{2}}{\sum_{i=1}^{n}{\left({\text{y}}_{\text{i}}-\bar{\text{y}}\right)}^{2}}$$

(4) $$RMSE=\sqrt{\frac{\sum_{i=1}^{n}{\left({\tilde{\text{y}}}_{\text{i}}-{\text{y}}_{\text{i}}\right)}^{2}}{nc}}$$

(5) $$RPIQ=\frac{Q3-Q1}{RMSE}$$

Where *y*_*i*_, $\tilde{y}{}_{i}$ are the measured PWC and predicted PWC of the *i*-th sample in different datasets; $\bar{y}$ is the mean value of PWC in the calibration and validation sets; and Q3 and Q1 are third quartile and first quartile of the datasets, respectively. According to the value of RPIQ, four categories of models are defined: RPIQ between 2.02 and 2.70 illustrates a poor model where only high and low values are distinguishable; RPIQ between 2.70 and 3.37 demonstrates a model where quantitative predictions are possible; RPIQ between 3.37 and 4.05 exhibits a good, quantitative model; and RPIQ > 4.05 implies an excellent model (Ludwig et al., 2017). Generally, a robust model should have a higher *R*^2^ and RPIQ and lower RMSE.

### Data Processing Software

Stepwise regression was analyzed in SPSS 19.0 (SPSS Inc., Chicago, United States). PLSR models were performed with PLS Toolbox version 6.20 (Eigenvector Research, Inc., Wenatchee, WA, United States) that ran under MATLAB version R2010a. Other band selection methods were performed with the same MATLAB environment. Graphs were prepared with Origin 9.0 (Microcal, United States).

## Results

### Variation of Winter Wheat Plant Water Content Between Water Treatments

**FIGURE 2:**
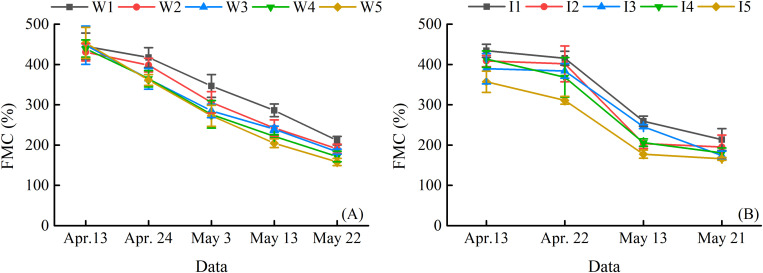
Winter wheat plant water content (PWC) at different growth stages in 2014–2015 **(A)** and 2015–2016 **(B)**. Vertical bars represent the standard deviation (SD) of PWC. W_1_, W_2_, W_3_, W_4_, and W_5_ represent the irrigation upper limits which were 80, 60, 45, 35, and less than 30% of the field capacity, respectively. I_1_ represents four irrigations at jointing, booting, flowering, and filling stage. I_2_ demonstrates three irrigations at jointing, booting, and filling stage. I_3_ shows two irrigations at jointing and flowering stage. I_4_ represents two irrigations at jointing and filling stage. I_5_ is the treatment without irrigation.

Figure 2 shows the PWC at the main growth stages in the 2014–2015 and 2015–2016 growing seasons. Winter wheat PWC tended to decrease throughout the entire growing seasons of two consecutive years, and the minimum PWC was obtained on May 22, 2015, and May 21, 2016, respectively. The PWC was increased with the increase of irrigation ceiling (2014–2015) and frequency (2015–2016).

### Correlation of PWC and Canopy Transformed Reflectance

**FIGURE 3:**
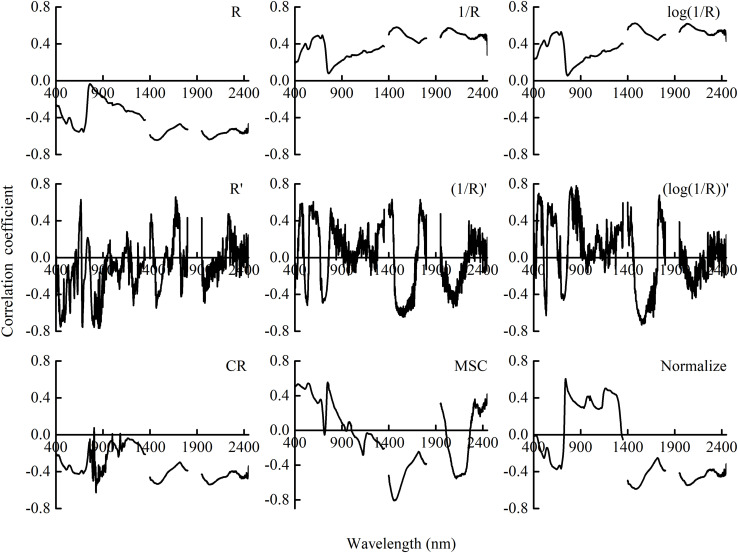
Correlation coefficient between PWC and canopy reflectance and its transformation in winter wheat. *R* is the raw reflectance of winter wheat. *R*′ is the first derivative of *R*. CR, MSC, and Normalize represent the transformations of raw reflectance by continuum removal, multiplicative scatter correction, and normalization, respectively.

The correlation coefficients between PWC and canopy reflectance with different transformation methods are shown in Figure 3. Correlations were negative in the range of the bands implemented in this study. Compared with raw canopy reflectance, curve with normalized transformation exhibited the same pattern and kept the basic features. 1/*R* and log(1/*R*) showed an opposite curve pattern compared with raw reflectance. The transformation methods with derivation can obviously enhance the correlation in the visible regions to raw reflectance, up to 0.76 for *R*′. The correlation coefficients for the treatment of CR significantly decreased. Multiplicative scatter correction (MSC) coefficients did exhibit the same pattern to *R*. The exception was that MSC was positive in the visible region.

### PLSR Model for Estimating PWC Based on the Full Spectrum

**FIGURE 4:**
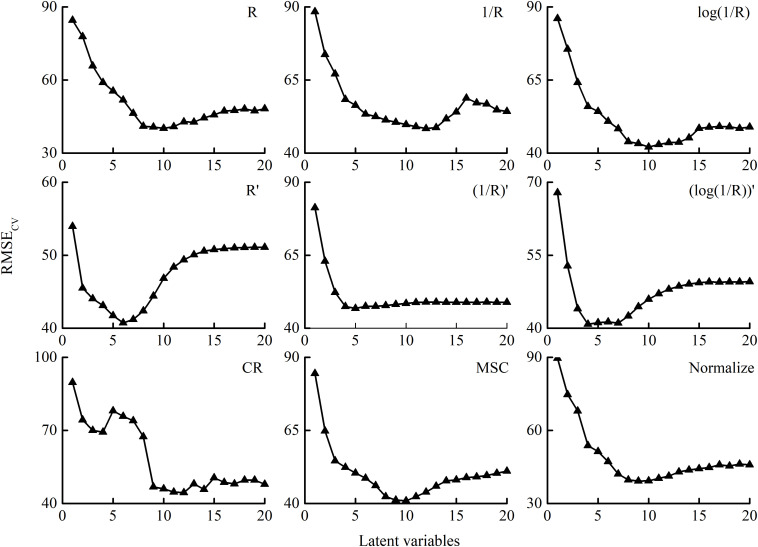
RMSE_CV_ of PLSR models with the number of latent variables (LVs) based on different transformation methods. *R* is the raw reflectance of winter wheat. *R*′ is the first derivative of *R*. CR, MSC, and Normalize represent the transformations of raw reflectance by continuum removal, multiplicative scatter correction, and normalization, respectively.

The RMSE trend of the PLSR model leave-one-out cross-validation based on different spectral transformation models is shown in Figure 4. Except for CR, the number of LVs for different transformation methods was selected by the minimum RMSE_CV_.

**FIGURE 5:**
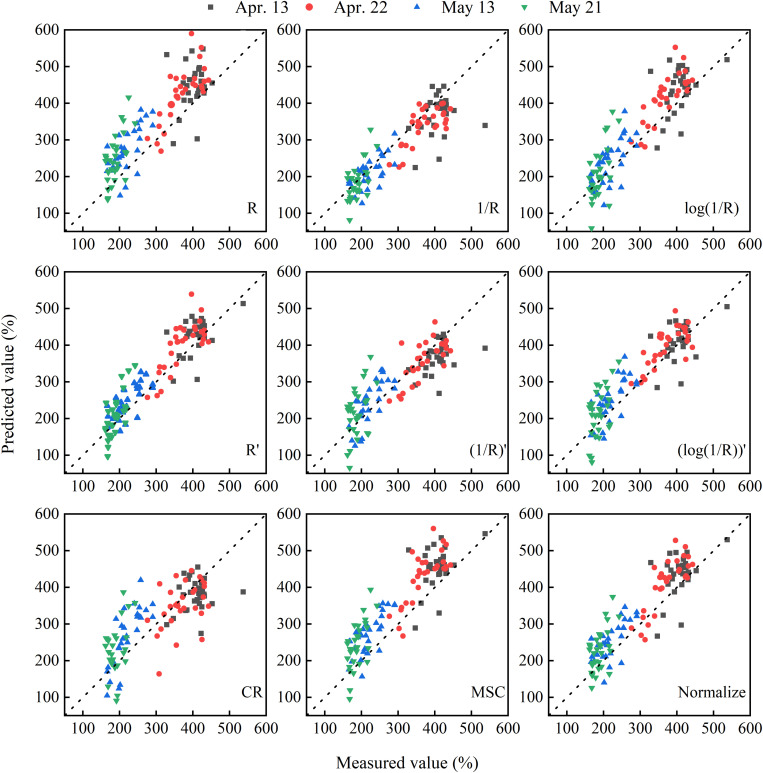
The correlation between measured and predicted values in estimating PWC in winter wheat based on different transformation methods. The dotted line represents the 1:1 line. *R* is the raw reflectance of winter wheat. *R*′ is the first derivative of *R*. CR, MSC, and Normalize represent the transformations of raw reflectance by continuum removal, multiplicative scatter correction, and normalization, respectively.

To quantify the PWC of winter wheat, the data from 2014 to 2015 through different pretreatments were used to establish the PLSR models. The data from 2015 to 2016 were used as an external validation to the PLSR models. The comparative analysis of the prediction accuracy of PLSR models based on different spectral transformations is listed in Table 3 and Figure 5. The results showed that the PLSR model based on the full spectrum was suitable for estimating the PWC with *R*^2^_C_ values above 0.87, except for the CR. The models based on the reciprocal method and the derivative method did show a higher *R*^2^_C_ (*R*^2^ > 0.90) and RPIQ_C_ (RPIQ > 6.0) and lower RMSE_C_ (RMSE < 31%). In addition, the models with derivative method (PLSR-4, PLSR-5, and PLSR-6) exhibited less LVs than the other treatments (except for CR). On the other hand, the performances of PLSR-5 and PLSR-6 in validation were poorer than PLSR-4, illustrating that the model was optimal. Therefore, among all the spectral transformation methods, the first derivative reflectance was superior in estimating the PWC in winter wheat.

**TABLE 3:** Performances of PLSR models for PWC based on different spectral transformation methods.

No.	Spectral transformations	Calibration dataset (2014–2015)					Validation dataset (2015–2016)		
		LVs	RMSE_CV_	*R*_C_^2^	RMSE_C_	RPIQ_C_	*R*_V_^2^	RMSE_V_	RPIQ_V_
1	*R*	8	40.99	0.88	35.35	5.33	0.78	75.03	2.68
2	1/*R*	12	48.38	0.91	30.78	6.12	0.82	47.81	4.20
3	log(1/*R*)	10	42.10	0.90	31.97	5.89	0.83	58.63	3.43
4	*R*′	6	40.73	0.96	20.49	9.19	0.86	46.27	4.34
5	(1/*R*)′	4	47.43	0.91	30.87	6.10	0.73	52.43	3.83
6	(log(1/*R*))′	4	40.84	0.91	29.95	6.29	0.81	50.54	3.98
7	CR	4	69.27	0.65	59.42	3.17	0.53	79.29	2.53
8	MSC	9	41.17	0.89	33.18	5.68	0.82	71.06	2.83
9	Normalize	8	39.67	0.88	34.70	5.43	0.83	56.64	3.55

*Latent variable indicates the number of latent variables selected by the model. R_C_^2^ and R_V_^2^ represent the coefficient of determination of model calibration and validation, respectively. RMSE_C_, RMSE_CV_, and RMSE_V_ represent the root mean square error of calibration, cross-validation (leave-one-out) and validation, respectively. RPIQ_C_ and RPIQ_V_ represent the ratio of performance to interquartile distance of the calibration and validation models, respectively. R is the raw reflectance of winter wheat. R′ is the first derivative of R. CR, MSC, and Normalize represent the transformations of raw reflectance by continuum removal, multiplicative scatter correction, and normalization, respectively.*

### PLSR Model for Estimating PWC Based on the Sensitive Bands

#### Selection of the Sensitive Bands

**FIGURE 6:**
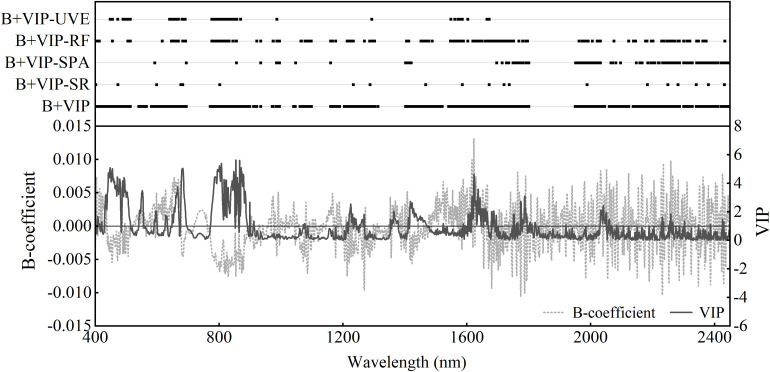
The distribution of sensitive bands with the first derivative transformation selected with different methods. Also shown are *B*-coefficient (gray line) and variable importance in projection (VIP) (black line) of the PLSR model based on the first derivative reflectance. B+VIP represents the joint analysis of regression coefficients and the variable importance in the projection of PLSR model. SR, SPA, RF, and UVE represent stepwise regression, successive projections algorithm, random frog, and uninformative variables elimination, respectively.

In order to simplify the model and avoid overfitting, sensitive bands were selected based on the first derivative transformed spectrum. The *B*-coefficients and VIP values derived from the PLSR-4 model indicated the importance of different bands in predicting PWC (Figure 6). The VIP and *B*-coefficients did show similar trends. First, bands where the VIP value was above 1 (the threshold of VIP) or the absolute values of *B*-coefficients were greater than the criterion (the mean of absolute value of *B*-coefficients) were selected. Due to the redundancy and inefficiency of the band, four methods (SR, SPA, RF, and UVE) were then used to further select the sensitive bands. The results of the method of using VIP and *B*-coefficient values of PLSR to select bands (denoted as B+VIP) and the four band selection methods are shown in Figure 6. B+VIP extracted the largest range of sensitive bands. In addition, other methods effectively reduced the number of sensitive bands, especially SR. The locations of the selected bands using different methods were similar, and they were located in the spectral regions centered at about 680, 860, 980, 1,285, 1,580, 1,660, 1,980, 2,184, 2,250, 2,350, and 2,430 nm. Most of the sensitive bands were distributed in the near-infrared and short-wave infrared regions.

#### PLSR Model Based on Sensitive Bands

**FIGURE 7:**
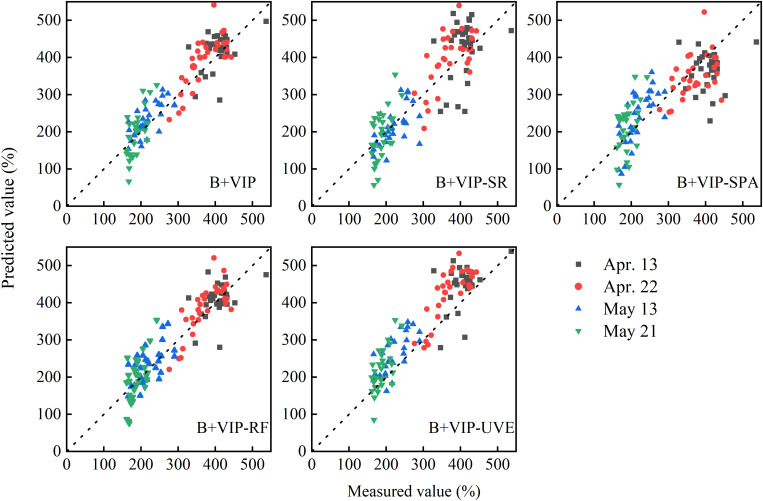
The correlation between measured and predicted values in estimating PWC in winter wheat using the sensitive bands with the first derivative transformation selected by SR in model validation. The dotted line represents the 1:1 line. B+VIP represents the joint analysis of regression coefficients and the variable importance in the projection of PLSR model. SR, SPA, RF, and UVE represent stepwise regression, successive projections algorithm, random frog, and uninformative variables elimination, respectively.

Using the sensitive bands selected in the *Selection of the Sensitive Bands* section, new PLSR models were established for estimating the PWC of winter wheat (Table 4), and the relationships between the measured value and the predicted value in model validation are shown in Figure 7. The models with sensitive bands demonstrated robust performance in model calibration (*R*^2^_C_ > 0.80, RMSE_C_ < 45%, and RPIQ_C_ > 4.2). Compared with the method (B+VIP of the PLSR model), the number of variables selected by the combined variable selection methods was reduced by at least 75%. Except for B+VIP-RF, the model accuracies were slightly reduced. Take B+VIP-SR for example, only 21 bands were selected to build the new model. The model accuracies were reduced in calibration and validation with *R*^2^ differences of 0.02 and 0.07 and RMSE differences of 4.22 and 15.3%, respectively. Among different variable selection methods, 244 sensitive bands were extracted through B+VIP-RF, and the model established by these sensitive bands performed best during model calibration and validation (*R*^2^_C_ = 0.99, RMSE_C_ = 11.53%, RPIQ_C_ = 16.34; *R*^2^_V_ = 0.84, RMSE_V_ = 44.40%, RPIQ_V_ = 4.52). Considering the number of variables and model performance, the combination of PLSR and RF (B+VIP-RF) was optimal. According to RPIQ_V_, the new model had good accuracy for PWC. In Figure 7, the data points were close to the 1:1 line, indicating good estimation in different ranges of PWC.

**TABLE 4:** The performance of PLSR models based on the first derivative spectrum sensitive bands.

Method	Number	Calibration dataset (2014–2015)			Validation dataset (2015–2016)		
		*R*_C_^2^	RMSE_C_	RPIQ_C_	*R*_V_^2^	RMSE_V_	RPIQ_V_
B+VIP	987	0.97	17.59	10.71	0.85	44.93	4.47
B+VIP-SR	21	0.95	21.81	8.64	0.78	60.23	3.34
B+VIP-SPA	145	0.81	44.67	4.22	0.62	63.89	3.14
B+VIP-RF	244	0.99	11.53	16.34	0.84	44.40	4.52
B+VIP-UVE	115	0.88	34.81	5.41	0.85	60.74	3.31

*B+VIP represents the joint analysis of regression coefficients and the variable importance in the projection of the PLSR model. SR, SPA, RF, and UVE represent stepwise regression, successive projections algorithm, random frog, and uninformative variables elimination, respectively. R_C_^2^ and R_V_^2^ represent the coefficient of determination of model calibration and validation, respectively. RMSE_C_, RMSE_CV_, and RMSE_V_ represent the root mean square error of calibration, cross-validation (leave-one-out) and validation, respectively. RPIQ_C_ and RPIQ_V_ represent the ratio of performance to interquartile distance of the calibration and validation models, respectively.*

## Discussion

### Methods for Spectral Proximal Sensing Data Preprocessing in Estimating Water Content

In the estimation with hyperspectral remote sensing, the disturbing factors (e.g., soil background and instrument noise) had a negative influence. By using suitable preprocessing and transformation, the accuracy and stability of the prediction model were improved. In this study, nine transformation methods were analyzed and compared. Compared with raw reflectance (*R*), the correlation between CR and PWC significantly decreased (Figure 3). The poor correlation with PWC was associated with the result of the transformation. The location and reflection feature of spectral absorption of variables is emphasized by CR transformation, and the values are normalized (Clark and Roush, 1984; Mutanga et al., 2005). However, the absorption features of parameters, in addition to water content, are also enhanced. It may result in the pattern of RMSE_CV_ fluctuated with the increase in the number of LVs (Figure 4). On the contrary, other transformation methods increased the correlation to some extent and did show good performance (*R*^2^_C_ > 0.87, RMSE_C_ < 36.00%, and RPIQ_C_ > 5.30) in model calibration. Consistent with Zhang et al. (2012), derivative transformations reduced the complexity of the model with less LVs but showed better correlation and good performance in water content estimation (Figure 4 and Table 3). The full-spectrum model with the first derivative transformation was the optimum (Table 3), indicating its advantages in estimating plant water content with canopy reflectance. Derivative transformation resolved overlapping spectra and emphasized the weak but meaningful peak (Shibayama et al., 1993). In this study, soil may contribute more to the canopy reflectance of plant under severe water stress. The first derivative transformation could suppress the spectral response of the soil background by converting it into a constant (Demetriades-Shah et al., 1990), and it could eliminate the effects of canopy architecture difference under various water conditions. In addition, our study elucidated that the correlation analysis can be used as an elementary selection method for spectral transformations.

### Approaches to Sensitive Band Selection

Five variable selection methods were used to extract the sensitive bands for PWC estimation. The numbers of input variables (sensitive bands) of the estimation models were significantly reduced, and B+VIP-SR had the most effects. The number of sensitive bands was reduced to only 21. Compared with the full spectrum, the calibration model with sensitive bands selected by B+VIP had higher estimation accuracy and better stability. This indicated that variable selection could reduce model complexity and improve model performance due to the removal of irrelevant and interference variables (Krishna et al., 2019). The *B*-coefficient and VIP value of the PLSR model reflected the weight of variables in predicting PWC, and they can be used as the basis for band selection (Sharabian et al., 2014; Wang et al., 2017). The criterion of the two parameters used in this study is effective to make it a good variable selection method. However, the number of selected bands is still very large, and further band extraction is needed. Thus, this method can be used as an initial band selection method.

On this basis, the other four methods were used to further reduce the number of sensitive bands. Comparing four combined band selection methods, the new calibration models demonstrated similar results, with high *R*^2^ and RPIQ and low RMSE. This indicated that the four methods used in this study were effective. Among the four methods, SPA showed the lowest *R*^2^ and the highest RMSE in model calibration, followed by UVE. It may be explained by the fact that SPA had selected the bands with minimum collinearity while ignoring some important bands and containing the noise information (Soares et al., 2013). Although SPA was performed on the basis of the PLSR (B+VIP) method, the noisy bands may still be selected. It indicated that B+VIP cannot remove all noisy bands. As for UVE, a previous study had proved that it can effectively extract sensitive bands (Yang et al., 2015). It determined invalid bands by comparing the stability coefficient of spectrum and noise. On the basis of PLSR, some effective bands with lower stability coefficient may be deleted, which resulted in the decreasing of model accuracy. It indicated that the combination of PLSR and UVE is not appropriate. With respect to the other methods, they showed good capabilities in band extraction. SR played a vital role in variable selection in a small number of samples, contributing to more reliable results. The result of PLSR-SR was similar to that of previous studies (Li et al., 2017; Wang et al., 2017; Xie et al., 2020), which reported that PLSR-SR could extract the effective sensitive bands and build the estimation model with acceptable accuracy. In addition, since only 21 bands were selected, PLSR-SR has the potential for developing the new instrument. RF can effectively extract sensitive bands (Table 4), which was proven by studies (Chen and Li, 2020). With 244 bands, PLSR-RF showed better or similar accuracy than PLSR in model calibration and validation, respectively. It indicated the advantages of RF in band extraction. Furthermore, the model accuracy has difference between the validation dataset and calibration dataset. In this study, two experimental datasets of different varieties and water stress treatments were used for model calibration and validation. Although the selected bands have good performance in model calibration, changes in cultivars and cultivation environment may cause the displacement of sensitive bands, resulting in some changes in the model validation. Considering the number of bands and the performance of model calibration and validation, PLSR-RF was the optimal method.

### Sensitive Bands for Plant Water Content Estimation

The sensitive bands selected through five variable selection methods did exhibit similar spectral regions with the central band being 680, 860, 980, 1,285, 1,580, 1,660, 1,980, 2,184, 2,250, 2,350, and 2,430 nm (Figure 6). It indicated that these central bands were useful in the estimation of winter wheat PWC. These sensitive bands were distributed in each spectral range studied. The new models based on the sensitive bands exhibited good estimation power with the RPIQ of model calibration and validation above 3.1. It is consistent with previous studies, showing that the combination of visible, near-infrared, and short-wave infrared bands certified robust performance (Zhang et al., 2013; Bendig et al., 2015; Kusnierek and Korsaeth, 2015). Using the method of B+VIP-RF, the estimation model had the best performance and the bands were located around 400–410, 500, 645–690, 770–850, 930, 980–995, 1,100, 1,165, 1,220, 1,290, 1,450–1,480, 1,550–1,590, 1,610–1,750, 1,960–1,990, 2,200, and 2,230–2,360 nm. It had been proved that the near-infrared and short-wave infrared spectral regions (900–2,500 nm) were directly affected by water (Gao and Goetz, 1994; Yebra et al., 2013). Majority of the sensitive bands were in the short-wave infrared region which may provide more information. Specifically, 980–995, 1,220, 1,450–1,480, 1,750, and 2,230–2,360 nm were near the major water absorption bands at 970, 1,200, 1,450, 1,750, and 2,250 nm (Danson et al., 1992; Shibayama et al., 1993; Pu et al., 2003; Clevers et al., 2008). The region of 1,150–1,260 nm was proved to be one of the optimal bands for ground-based remote sensing of vegetation water content (Sims and Gamon, 2003). The 2,230–2,360-nm bands were located in the region of 2,080–2,350 nm which can be used for water content estimation, but it was strongly affected by the soil reflectance when the cover is less than 100% (Ripple, 1986). Concurrently, many selected sensitive bands were indirectly relevant to the plant water content. The selected bands located in the visible region were related to the absorption of chlorophyll content in plants (Yebra et al., 2013; Steidle Neto et al., 2017). Band valleys around 500 and 680 nm did show a good correlation with the chlorophyll pigments representing the color characteristics (Yu et al., 2014). This can be explained by that water content exhibited a significant correlation with chlorophyll content and other pigments (Jaleel et al., 2009; Keyvan, 2010; Din et al., 2011). Some bands were located in the near-infrared region, which reflected the internal structure of the cells. It is mainly because plant water status directly influences cell turgor and internal space of the tissue resulting in the change of internal scattering. Some bands were selected in the specific region which were associated with the absorption of protein for the N–H asymmetry stretch and amide II at 1,980 nm and C–H stretch at 2, 240 nm (Curran, 1989; Sandra et al., 2000). Presumably, it is because of the close relationship between water status and protein.

### Estimation of Plant Water Content With Multivariate Statistical Methods

As a powerful multivariate statistical method, PLSR models had good performance in estimating the PWC of winter wheat (Tables 3, 4). In model validation, the data distributions from different growth stages were inconsistent (Figures 5, 7), indicating that model performance was affected by plant growth stages. The results were in accordance with previous studies (Rischbeck et al., 2016; Wang et al., 2017). Plant development was related to plant internal structure, substances, and function, which would affect the location of sensitive bands in different growth stages. As the growth period is promoted, the data points of majority models were getting closer to the 1:1 line, and the RMSE of the measured and predicted PWC decreased (data not shown), with the minimum RMSE appearing on May 21. It indicated that the filling stages performed better than the other stages. In addition, the model performance was not only affected by the plant growth but also by the cultivars and experimental treatment (El-Hendawy et al., 2019a, b). In this study, the influence of these factors on multivariate statistics was not considered, and further research would be conducted in the future.

## Conclusion

In this study, the results demonstrated that the canopy spectral proximal sensing data could be used for estimating the PWC of winter wheat in an accurate and nondestructive way. Among different spectral transformation methods, the first derivative transformation had closer correlation with PWC, and the PLSR-4 model was more effective in calibration and validation, indicating that the first derivative transformation was the optimal method for processing the canopy spectral data in estimating PWC. The sensitive bands extracted by the combination of PLSR and RF obtained the best performance. The verification accuracy of the model slightly decreased compared with the full-spectrum model, which is acceptable. The comprehensive method (PLSR-RF) performed best in extracting the sensitive bands and building a simple and stable model. The findings of this study provide technical support for large-scale monitoring of plant water status by using canopy spectral proximal sensing data in field production.

## Data Availability Statement

The original contributions presented in the study are included in the article/supplementary material, further inquiries can be directed to the corresponding author/s.

## Author Contributions

HS, MF, and WY conceived the research. HS, MF, LX, and WY designed the experiments. HS, LX, CW, XJ, GW, and SZ carried out the field measurements. HS, MF, and CW provided technical support. GD reviewed and edited the manuscript. All authors have read and agreed to the published version of the manuscript.

## Conflict of Interest

The authors declare that the research was conducted in the absence of any commercial or financial relationships that could be construed as a potential conflict of interest.

## Abbreviations

C: calibration
CV: cross-validation
FC: field capacity
LV: latent variable
MSC: multiplicative scatter correction
PLSR: partial least squares regression
PWC: plant water content
*R*^2^: coefficient of determination
RF: random frog
RMSE: root mean square error
RPIQ: ratio of performance to interquartile distance
SD: standard deviation
SPA: successive projections algorithm
SR: stepwise regression
UVE: uninformative variables elimination
V: validation
VIP: variable importance in projection.
